# Viral Infection and Stress Affect Protein Levels of Dicer 2 and Argonaute 2 in *Drosophila melanogaster*

**DOI:** 10.3389/fimmu.2020.00362

**Published:** 2020-03-04

**Authors:** Alessandro Torri, Vanesa Mongelli, Juan A. Mondotte, Maria-Carla Saleh

**Affiliations:** Viruses and RNA Interference Unit, CNRS Unité Mixte de Recherche, Institut Pasteur, Paris, France

**Keywords:** RNA interference, insect immunity, gene regulation, viral infection, antiviral response, protein regulation

## Abstract

The small interfering RNA (siRNA) pathway of *Drosophila melanogaster*, mainly characterized by the activity of the enzymes Dicer 2 (Dcr-2) and Argonaute 2 (Ago-2), has been described as the major antiviral immune response. Several lines of evidence demonstrated its pivotal role in conferring resistance against viral infections at cellular and systemic level. However, only few studies have addressed the regulation and induction of this system upon infection and knowledge on stability and turnover of the siRNA pathway core components transcripts and proteins remains scarce. In the current work, we explore whether the siRNA pathway is regulated following viral infection in *D. melanogaster*. After infecting different fly strains with two different viruses and modes of infection, we observed changes in Dcr-2 and Ago-2 protein concentrations that were not related with changes in gene expression. This response was observed either upon viral infection or upon stress-related experimental procedure, indicating a bivalent function of the siRNA system operating as a general gene regulation rather than a specific antiviral system.

## Introduction

RNA interference (RNAi) is a defensive and gene regulatory process based in sequence homology among nucleic acids (1–3). Small RNAs (sRNAs) are produced and used as guides to target complementary DNA or RNA sequences (4, 5). Three main sRNA pathways are described to date, differing in the origin and biogenesis of the double-stranded sRNAs and their molecular function: the micro RNA pathway (miRNA), the small interfering RNA pathway (siRNA), and the Piwi-interacting RNA pathway (piRNA) (6).

In the siRNA pathway, the ribonuclease Dicer 2 (Dcr-2) recognizes and dices double stranded RNA molecules of exogenous (virus) or endogenous (cellular) origin, producing 21-nucleotide length siRNA duplexes that are loaded into the protein Argonaute 2 (Ago-2) within the RNA-induced silencing complex (RISC). Once loaded, the siRNA duplexes are unwound and only one RNA strand is used by Ago-2 to target and slice the complementary RNA. Two Dcr-2 cofactors are indispensable for siRNA production and correct loading into RISC: LOQS and R2D2 (4, 6).

The siRNA pathway is considered the most ancient and at the origin of the RNAi phenomenon. Its main components (Argonaute-Piwi, Dicer-like, and RNA-dependent RNA Polymerase proteins) are supposed to have been already present in the last common ancestor of eukaryotes (7). In *Drosophila melanogaster* the siRNA pathway acts as the main antiviral immune response (8) and is also involved in somatic defense against transposons (9). However, the fact that several siRNAs target cellular mRNAs (7, 10) and repress the expression of specific genes (11), suggests that it may also play a role in the regulation of gene expression.

Much effort has been dedicated to studying the RNAi mechanism and pathways, but despite several advancements, not much is known about their regulation (12). Previous studies reported that the mRNA expression of the core components of the siRNA pathway (Dcr-2 and Ago-2 among others) are induced by double stranded RNA (dsRNA) in *Acyrthosiphon pisum* (13), *Manduca sexta* (14), and *Blatella germanica* (15) through a yet unknown mechanism. This induction has also been shown upon viral infection in *Bombus terrestris* (16), *Apis mellifera* (17) and *D. melanogaster* (18). However, in *Apis mellifera* this phenomenon seems to be virus-specific (19) and in *D. melanogaster* the induction was observed upon injection of Zika virus, an arbovirus for which the fruit fly is not a natural host (18). In addition, a study in *D. melanogaster* (20) showed increased levels of *Ago-2* and *Dcr-2* in flies constitutively expressing an active form of dFOXO, establishing a link between stress response and RNAi regulation. However, knowledge on regulation, stability, and turnover of the siRNA pathway core genes and proteins remains scarce.

Here we explore whether the siRNA pathway is regulated at the transcriptional and/or at the translational level following viral infection in *D. melanogaster*. We analyzed the expression of transcripts and proteins for *Dcr-2* and *Ago-2* in three different fly strains infected with Drosophila C Virus (DCV) and Flock House Virus (FHV) by two different modes of delivery, injection and oral infection. Our results show a complex and previously undescribed mechanism of regulation of the siRNA pathway at the protein level independent of fly strain, gene expression and mode of infection.

## Materials and Methods

### Fly Strains and Husbandry

The *D. melanogaster* fly lines used were the following: *w*^1118^, *Oregon-R*, and *yw*. Fly stocks harbor the sensitive allele of Pastrel 3L:7350895 (Thr). Flies were reared on a standard cornmeal diet (Bloomington) at a constant temperature of 25°C and kept under a 12:12 photoperiod. All fly lines were cleaned of possible chronic infections (viruses and *Wolbachia*). In addition, fly stocks were analyzed by RT-PCR with pairs of primers specific for CrPV, DAV, DXV, DCV, FHV, and NoraV to confirm that they were not persistently infected by these viruses.

### Virus Production and Titration

DCV stock was prepared in *w*^1118^ flies. Flies were injected intrathoracically with 500 TCID50 per fly. When mortality started, flies were anesthetized and squashed in PBS (3 flies per 100 μl of PBS). The extract was frozen at −80°C, thawed and centrifuged for 15 min at 15,000 × g at 4°C. The supernatant was recovered and filtered to eliminate bacteria, aliquoted, and stored at −80°C.

FHV stock was prepared on low-passage S2 cells. When the cytopathic effect started, the supernatant was harvested and centrifuged.

Both stocks were titrated in S2 cells. Titers were measured by end-point dilution method and expressed as 50% Tissue culture Infective Dose (TCID_50_). DCV stock: 1,18 × 10^10^ TCID_50_/ml, FHV stock: 5 × 10^9^ TCID_50_/ml).

### Viral Infections

Injections: flies were injected intrathoracically using a nanoject (Nanoject II apparatus; Drummond Scientific) with 50 nL of a viral suspension of 10 TCID_50_ of Drosophila C virus or 100 TCID_50_ of Flock house virus in 10 mM Tris, pH 7. An injection of the same volume of 10 mM Tris, pH 7 served as a mock-infected control. Infected flies were kept at 25°C and changed to fresh vials every 2 days.

Oral infections: flies were starved during 5 h in an empty tube. Then, flies were transferred to a tube containing a Whatman filter paper in the bottom embedded in a mix of viral stock in PBS (10% viral stock, 35% sucrose and 2% of blue dye). After 16 h, only the flies having a blue-belly (corresponding to blue dye in the gut due to ingestion) were placed in new media tubes, kept at 25°C and changed to fresh vials every 2 days.

### General Experimental Design

For all the experiments, 4- to 7-day old adult female flies were used. For each experimental condition, flies were divided in two pools: one pool was used for viral titration and RNA extraction, and the other one for protein extraction. For every experiment presented, the analyses were based on three biological replicates. In **Figure 2**, 16 biological replicates were performed (except *w*^1118^ in **Figure 2B**, *n* = 15). For western blots, only biological replicates were used. For RT-qPCR, three technical replicates per condition and per biological replicate were used.

### Survival Assays

Mortality of infected flies was measured daily by counting the number of dead flies in each test tube. Three biological replicates of 60 flies each were done per condition. Fly mortality at day 1 was attributed to damage invoked by injection and/or manipulation procedure, and excluded from further analyses.

### RNA Extractions and RT-qPCR

For each time point and condition, total RNA was extracted from a pool of 4–12 flies depending on the biological replicate. Each pool was homogenized in 300 μl of PBS and 100 μl were used to perform RNA extractions using TRIzol reagent (Invitrogen).

The first-strand cDNAs were produced from 400 ng of RNA using Maxima H Minus First Strand cDNA Synthesis Kit with dsDNase (Thermo Scientific) according to the manufacturer's instructions. For each sample, a negative control without the reverse transcriptase enzyme was performed, in order to check for potential genomic DNA contamination. Roche Universal Sybr Green Master Mix (Rox) was used for qPCR. The sequences of the primers used were:

*Ago-2* F primer: 5′-GTGGTTTACACGCCTCCTCA-3′

*Ago-2* R primer: 5′-GGGTAGTTGCGACTGTGGAA-3′

*Dcr-2* F primer: 5′-GGGTGAACAGGGAGTGGATG-3′

*Dcr-2* R primer: 5′-CAAAAAGACCTGGGCTGTGC-3′

Quantification was normalized to that of mRNA encoding the endogenous ribosomal protein Rp49 as previously reported (21). Data were calculated using the ΔΔCt method to compute relative gene expression. For each sample, 3 technical replicates plus 1 RT negative control were included in the qPCR plate. qPCR was performed in 384-well plates with a final volume of 10 μl with QuantStudio 7 Flex Real-Time PCR System (Applied Biosystems). The following program was used: Hold stage 50°C for 2 min, 95°C for 10 min. PCR stage: 40 cycles of 95°C for 15 s, 60°C for 1 min. A melt curve to confirm the specificity of the reaction was performed.

### Protein Extraction, Western Blot, and Protein Quantification

For each time point and condition, total proteins were extracted from pools of 4–8 flies, depending on the biological replicate, using 200 μl NP40 Buffer: 20 mM HEPES-KOH buffer pH 7.5; 100 mM KCl; 5% Glycerol; 0.05% NP40; 1 mM DTT (freshly added) and 1x complete, EDTA-Free Protease Inhibitor Cocktail (Roche) (freshly added). Each pool was homogenized with a pestle, centrifuged at 12,000 × g for 10 min and the supernatant was recovered and stored at −80°C. Ten microliters of each protein extract were ran in SDS-PAGE using Mini-PROTEAN TGX Stain-free gels 4–20% (BIO-RAD) according to the manufacturer's instructions. The gels were then activated for image acquisition using Molecular Imager Gel Doc XR+ (BIO-RAD) and the transfer was performed using Trans-Blot Turbo Transfer Pack nitrocellulose membranes and Trans-Blot Turbo Transfer System (BIO-RAD). The image acquisition of the total amount of proteins transferred was performed with Molecular Imager Gel Doc XR+ (BIO-RAD). For the immunoblots, the following primary antibodies were used: Anti-Dcr-2 Abcam AB4732 (1:1000); Anti-Ago-2 Siomi 9D6 (non-commercial antibody, 1:15) kindly provided by Haruhiko Siomi. The secondary antibodies were HRP-linked: anti-rabbit GE Healthcare NA9340V (1:10000); anti-mouse Abcam AB6728 (1:10000). The antibodies were diluted in a solution of PBS-BSA 3%, TWEEN 20 0.3%. Membranes were incubated overnight at 4°C with the primary antibodies, washed 3 times for 5 min with PBS-TWEEN 20 0.3%, incubated with secondary antibodies 1 h at room temperature and washed again 3 times for 5 min with PBS-TWEEN 20 0.3% before adding the ECL (SuperSignal West Pico Plus Chemiluminescent substrate—Thermo Scientific). The image acquisitions were performed with MyECL Imager (Thermo Scientific). The intensities of the bands corresponding to Dcr-2 and Ago-2 were normalized with the total amount of protein in their respective lanes using ImageStudioLite (LI-COR Biosciences) (Supplementary Figures 1, 2).

In order to compare results from different experiments, two types of normalization were employed. To study differences in the relative protein expression, all the data were normalized with the mock-infected time point 0 (**Figures 5**, **6**). To analyze non-relative protein levels, all data were corrected for the experimental effect as previously shown (21). This procedure transforms the raw values into their deviation from the experimental mean, and the resulting adjusted values are centered on zero (**Figures 2**, **7**).

### Statistical Analysis

All statistical analyses were performed with Prism 8—GraphPad. The following statistical tests were used: for grouped tables with two grouping variables, two-way ANOVA followed by Sidak's multiple comparisons test; for table with one grouping variable, the Mann-Whitney test; for survival curves, the log-rank (Mantel-Cox) test; to calculate the probability of deviations from a theoretically expected distribution, the binomial test.

## Results

### Different *D. melanogaster* Strains Differ in Their Susceptibility to Viral Infections

**Figure 1:**
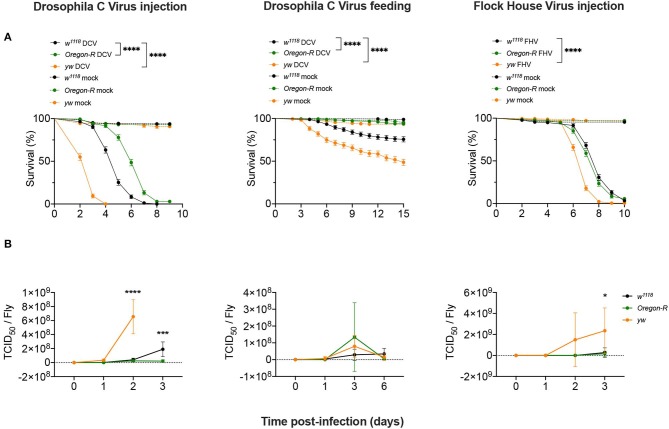
Susceptibility to viral infections. **(A)** *w*^1118^, *Oregon-R* and *yw* adult flies were intrathoracically injected with 10 TCID_50_ of DCV (Left panel), 100 TCID_50_ of FHV (Right panel), or orally infected with a solution of sucrose and blue dye containing 1.18 × 10^10^ TCID_50_ of DCV (Middle panel). Mock controls were injected or fed with Tris buffer. Survival was determined daily. Each curve represents three independent biological experiments of 60 flies each. Survival curves were compared by log-rank (Mantel-Cox) test (****P < 0.0001). **(B)** *w*^1118^, *Oregon-R* and *yw* viral titers over time. Each time point represents mean and SD of three different biological replicates composed of a pool of 4–12 flies. The infection conditions were the same as described in **(A)**. Controls were negative and are not shown in the figure. Each time point was compared to each other by two-way ANOVA followed by Sidak's multiple comparisons test (*P < 0.05; ***P < 0.0002).

To study the regulation of the siRNA pathway during viral infections we used different *D. melanogaster* strains, viruses and modes of infection in order to establish the commonality of the response. We challenged *w*^1118^, *Oregon-R*, and *yw D. melanogaster* strains in two different infection conditions: (1) injection of Drosophila C Virus [DCV; (+)ssRNA *Dicistroviridae*], a natural Drosophila pathogen; and of Flock House Virus [FHV; bisegmented (+)ssRNA, *Nodaviridae*], a non-natural fly pathogen used as a control; and (2) oral infection by feeding with DCV. Survival curves showed that after DCV infection by injection, *w*^1118^ flies had an intermediate survival rate compared to *yw* flies that were more susceptible, whereas *Oregon-R* were more resistant (Figure 1A). Injection of DCV resulted in the death of all flies at 8 days post-infection (dpi) for *w*^1118^ and 4 dpi for *yw* flies, while 97% of *Oregon-R* flies died at 9 dpi (Figure 1A, left panel). In flies orally infected with DCV, the proportions of mortality 15 days after infection were of ~25% for *w*^1118^, ~50% for *yw*, and ~6% for *Oregon-R* strains (Figure 1A, middle panel). Upon FHV injection, *yw* were still the most susceptible flies, with ~35% mortality at day 6 compared with ~9% for *w*^1118^ and ~15% for *Oregon-R* (Figure 1A, right panel). We then measured viral loads by TCID_50_ for all tested conditions at 0 dpi (15 min post-injection or 16 h post-feeding) and at 3 further times post-infection (Figure 1B and Supplementary Table 1). Following virus injection, both DCV and FHV reached higher titers in *yw* flies than *w*^1118^ and *Oregon-R* flies at 2 dpi and 3 dpi, respectively, while DCV accumulated to higher levels at 3 days post-injection in *w*^1118^ flies compared with *Oregon-R* (Figure 1B and Supplementary Table 1). However, no significant differences in DCV titers were found between the different fly strains during oral infections (Figure 1B and Supplementary Table 1). Taken together, these results show that these fly strains differ in their susceptibility to viral infections and that there is a correlation between the increase of viral loads and mortality (Figure 1B and Supplementary Table 1), indicating that flies are dying due to the physiological burden imposed by the infection.

### Basal Levels of Dcr-2 and Ago-2 Proteins Do Not Account for the Variation in Susceptibility to Viral Infection

**Figure 2:**
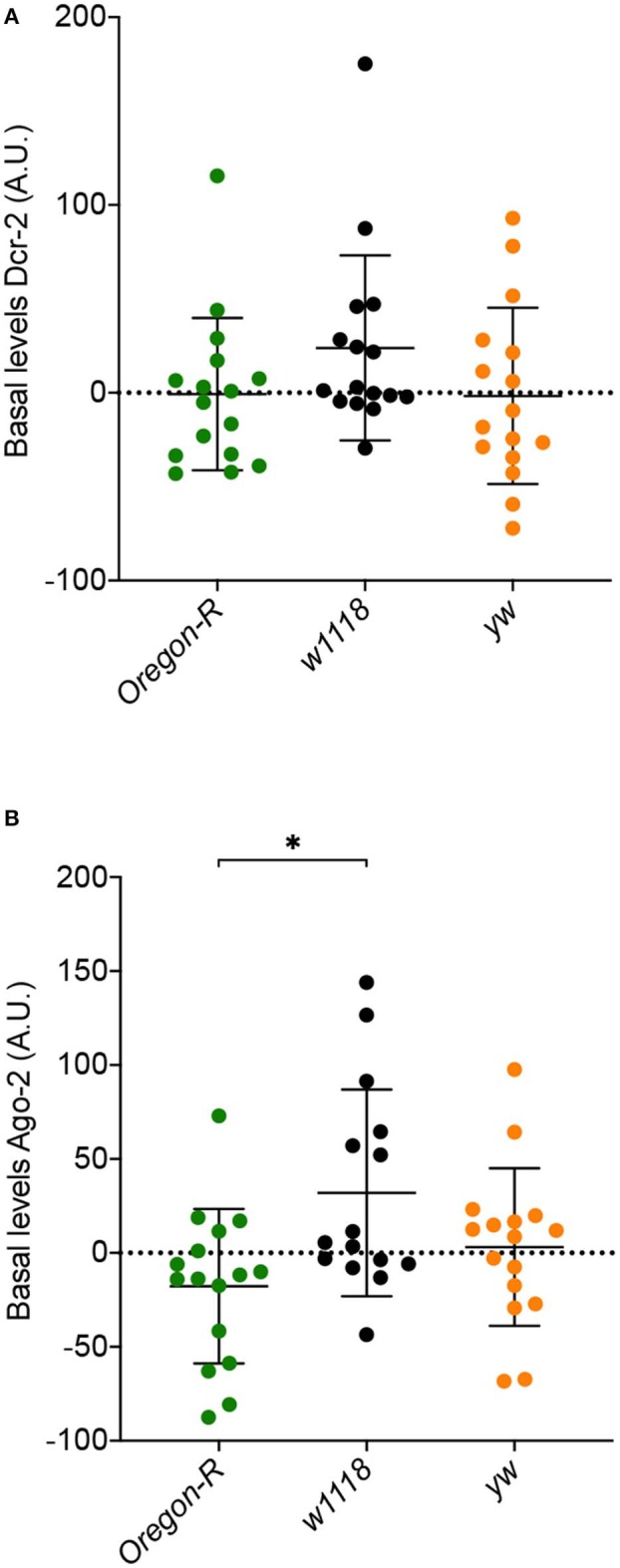
Basal expression levels of core RNAi proteins in *Oregon-R, w*^1118^, and *yw* flies determined by western blot (quantification and normalization are detailed in Materials and Methods). **(A)** Dcr-2 basal levels. **(B)** Ago-2 basal levels. Each dot represents a biological replicate composed of a pool of 5 flies. A.U. indicates arbitrary units. *Indicates significant differences among *Drosophila* strains using Mann–Whitney test (*P* = 0.0106).

We next asked if the differences in susceptibility and viral accumulation observed between *w*^1118^, *Oregon-R* and *yw* flies might be due to differences in the basal levels of the siRNA pathway core proteins, Dcr-2 and Ago-2, before viral infection. To address this question, we measured the levels of these proteins in the three Drosophila strains by western blot. Dcr-2 basal levels were similar between strains (Figure 2A). A significant increase in Ago-2 basal levels between *w*^1118^ and *Oregon-R* (Figure 2B) was observed, but it cannot explain the difference in susceptibility to virus infection observed for these strains. Taken together, these results show that the basal levels of Dcr-2 and Ago-2 do not play a critical role in fly survival and in the control of viral infections.

### Viral Infection Does Not Change *Dcr-2* and *Ago-2* Gene Expression Levels

**Figure 3:**
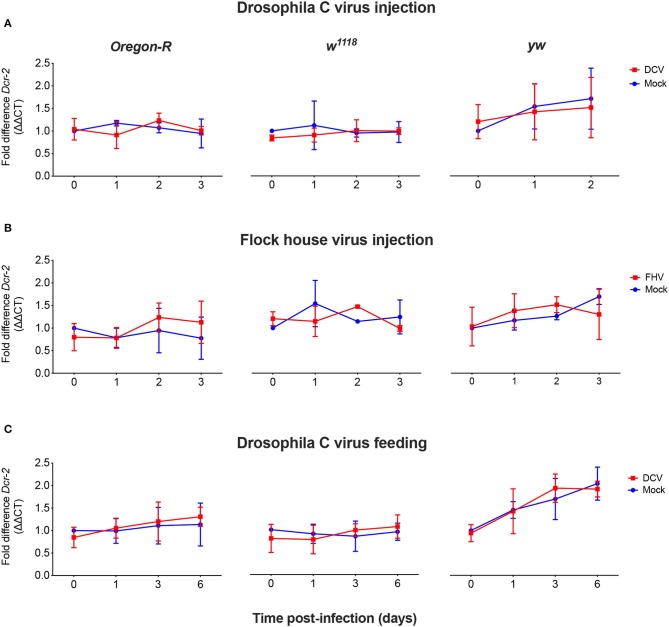
Relative *Dcr-2* mRNA expression levels after viral infection. *Oregon-R, w*^1118^, and *yw* adult flies were intrathoracically injected with 10 TCID_50_ of DCV **(A)**, 100 TCID_50_ of FHV **(B)**, or orally infected with a solution of sucrose and blue dye containing 1.18 × 10^10^ TCID_50_ of DCV **(C)**. Mock controls were injected or fed with Tris buffer. Numbers in x-axis represent days post-infection. Time point 0 corresponds to 15 min post-injection in **(A,B)** and 16 h post-feeding in **(C)**. Each curve represents three biological independent experiments with three technical replicates each. In each condition and for each time point, the cDNA from pools of 4–12 flies was used to quantify the expression levels of *Dcr-2* by qPCR. Each data was normalized with the time point 0 of the mock infected. No statistical significance in *Dcr-2* transcript accumulation due to viral infection was found. *n* = 3, mean ± SD, two-way ANOVA followed by Sidak's multiple comparisons test.

**Figure 4:**
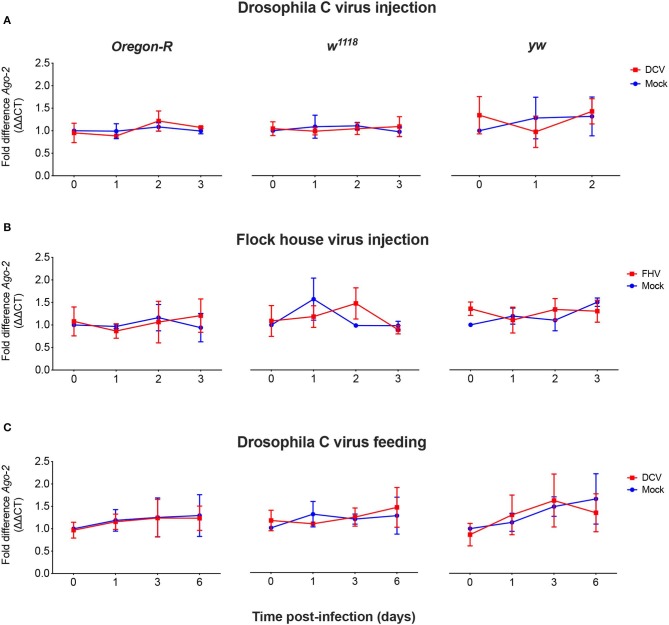
Relative *Ago-2* mRNA expression levels after viral infection. *Oregon-R, w*^1118^ and *yw* adult flies were intrathoracically injected with 10 TCID_50_ of DCV **(A)**, 100 TCID_50_ of FHV **(B)**, or orally infected with a solution of sucrose and blue dye containing 1.18 × 10^10^ TCID_50_ of DCV **(C)**. Mock controls were injected or fed with Tris buffer. Numbers in x-axis represent days post-infection. Time point 0 corresponds to 15 min post-injection in **(A,B)** and 16 h post-feeding in **(C)**. Each curve represents three biological independent experiments with three technical replicates each. In each condition and for each time point, the cDNA from pools of 4–12 flies was used to quantify the expression levels of *Ago-2* by qPCR. Each data was normalized with the time point 0 of the mock infected. No statistical significance in Ago-2 transcript accumulation due to the viral infection was found. *n* = 3, mean ± SD, two-way ANOVA followed by Sidak's multiple comparisons test.

As we did not observe a correlation between Dcr-2 and Ago-2 proteins basal levels and infection outcomes in the *D. melanogaster* strains tested, we wanted to explore if a difference in gene expression would be noticeable as a result of the viral infection. We evaluated the mRNA expression levels of *Dcr-2, Ago-2*, and *rp49* (housekeeping gene) by qPCR at four time points post-infection. The mRNA was extracted from the same pools of flies used in Figure 1B. We did not observe any significant difference in *Dcr-2* (Figure 3) and *Ago-2* (Figure 4) mRNA levels of infected flies compared with mock infected flies, independent of the fly strain, virus or mode of infection used.

### Viral Infection Induces an Immediate Change in Dcr-2 and Ago-2 Protein Levels

**Figure 5:**
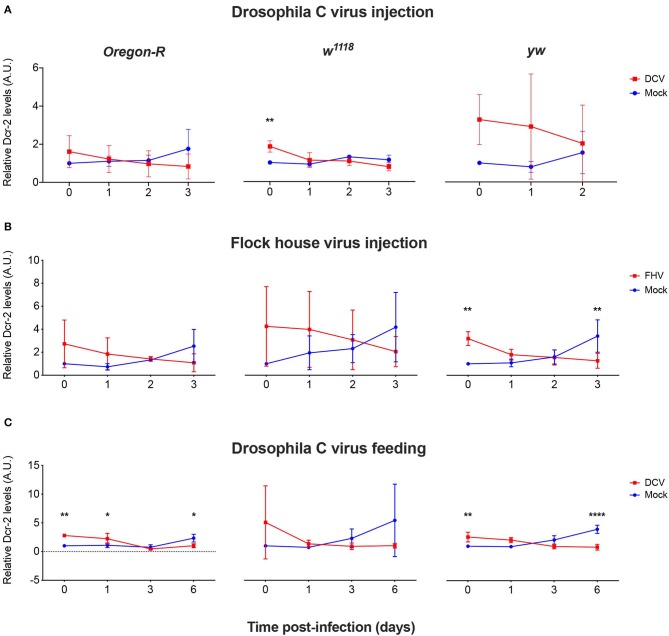
Relative Dcr-2 protein expression levels after viral infection. *Oregon-R, w*^1118^, and *yw* adult flies were intrathoracically injected with 10 TCID_50_ of DCV **(A)**, 100 TCID_50_ of FHV **(B)**, or orally infected with a solution of sucrose and blue dye containing 1.18 × 10^10^ TCID_50_ of DCV **(C)**. Mock controls were injected or fed with Tris buffer. Numbers in x-axis represent days post-infection. Time point 0 corresponds to 15 min post-injection in **(A,B)** and 16 h post-feeding in **(C)**. Each curve represents three independent biological experiments. In each condition and for each time point, total protein extraction from pools of 4–8 flies was used to quantify the expression levels of Dcr-2 by western blot. Each band corresponding to Dcr-2 was normalized with the total amount of protein of its lane and each set of data was normalized with the time point 0 of the negative control. Relative levels of Dcr-2 in infected flies at time point 0 were higher compared with the control. A.U. indicates arbitrary units. *n* = 3; mean ± SD, two-way ANOVA followed by Sidak's multiple comparisons test (*P < 0.05, **P < 0.01, ****P < 0.0001).

**Figure 6:**
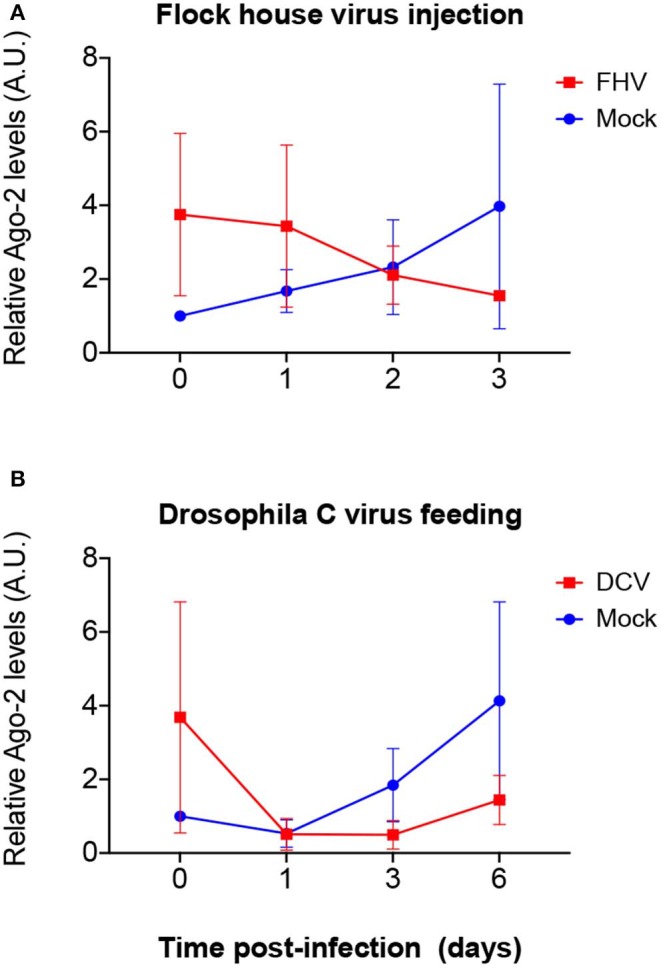
Relative Ago-2 protein expression levels after viral infection. *w*^1118^ adult flies were intrathoracically injected with 100 TCID_50_ of FHV **(A)** or orally infected with a solution of sucrose and blue dye containing 1.18 × 10^10^ TCID_50_ of DCV **(B)**. Mock controls were injected or fed with Tris buffer. Numbers in x-axis represent days post infection. Time point 0 corresponds to 15 min post-injection in **(A)** and 16 h post-feeding in **(B)**. Each curve represents three independent biological experiments. In each condition and for each time point, the total protein extract from pools of 4–8 flies was used to quantify the expression levels of Ago-2 by western blot. Each band corresponding to Ago-2 was normalized with the total amount of protein of its lane and each set of data was normalized with the time point 0 of the negative control. Relative levels of Ago-2 in infected flies at time point 0 were higher compared with the control but without statistical significance. A.U. indicates arbitrary units. *n* = 3; mean ± SD; two-way ANOVA followed by Sidak's multiple comparisons test.

To explore if mRNA expression levels correlate with proteins levels upon viral infection, we quantified the expression of Dcr-2 and Ago-2 proteins. Proteins were extracted from flies that were biological replicates to Figures 1B, 3, 4 and the presence and quantity of Dcr-2 and Ago-2 were analyzed by western blot. After normalization with the time point 0 of the mock-infected condition (see Materials and Methods for details on normalization protocol and Supplementary Figures 1, 2), Dcr-2 levels showed a relative increase in virus infected flies at the time point 0 (15 min post-injection or 16 h post-feeding) independently of the fly strain, virus and mode of infection used (Figure 5). These increments were statistically significant for *w*^1118^ injected with DCV (Figure 5A), for *yw* injected with FHV (Figure 5B), and for *Oregon-R* and *yw* orally infected with DCV (Figure 5C). These significant increases in Dcr-2 protein expression were not observed throughout all the conditions; however, in the 27 western blots performed (3 biological replicates × 3 fly strains × 3 different modes of infection), 26 of them showed higher Dcr-2 levels in infected flies than in the mock infected control. The probability that this phenomenon is due to a random effect is only 0.00002% (binomial test assuming that, during a random phenomenon, the probability to be higher or lower with respect to the control is the same). On the contrary, at the last time point (3 days post-infection for injection and 6 days post-infection for feeding) we found that Dcr-2 protein relative levels in infected flies were lower compared to the mock-infected flies. This decrease was statistically significant for *yw* injected with FHV (Figure 5B), and for *Oregon-R* and *yw* orally infected with DCV (Figure 5C). In injected flies, we found this pattern in 14 of 15 experiments with a probability of 0.004% for this phenomenon to be due to a random effect. In orally infected flies, we found this pattern in 9 of 9 experiments, with a probability of 0.2%. Due to the limited amount of anti-Ago-2 antibody in our possession (non-commercial antibody), we chose to perform the analysis for Ago-2 protein levels only in the *w*^1118^ strain. We observed the same trend as for Dcr-2, but without significant differences (Figure 6). From a total of 7 western blots performed, we found higher relative levels of Ago-2 7 times for the time point 0, with respect to mock infected flies, with a probability of 0.78% (binomial test) that this is due to a random effect.

Altogether, the results show that Dcr-2 and Ago-2 protein levels change promptly upon virus infection.

### Viral Presence, as Well as Infection Procedure Related-Stress, Induce a Change in Dcr-2 Protein Accumulation

**Figure 7:**
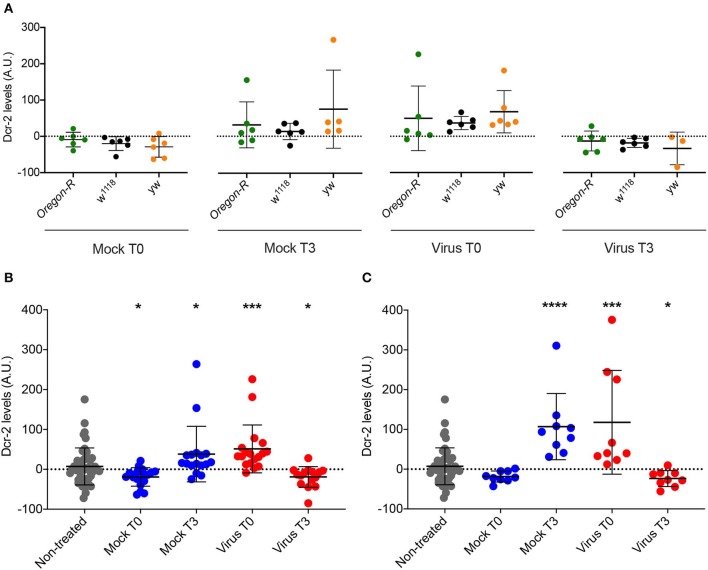
Comparison of Dcr-2 protein expression levels. **(A)** Dcr-2 levels for 2 time points after mock and virus infection. For each time point, Dcr-2 levels were compared between *Oregon-R, w*^1118^, and *yw* flies (Virus indicates flies injected with DCV or FHV). Each dot indicates a biological replicate composed of a pool of 4–8 flies. No significant differences were observed between the strains inside each time point. Mean ± SD are represented. Analysis performed using the Mann–Whitney test, *n* = 6 except *yw* Mock T3 *n* = 5 and *yw* Virus T3 *n* = 3. **(B)** Dcr-2 levels along time (days post injection); Mock T0 = Mock-control at time point 0; Mock T3 = Mock-control at time point 3; Virus T0 = Virus infection at time point 0; Virus T3 = Virus infection at time point 3. Analysis performed using the Mann–Whitney test; all groups are compared with non-treated. Non-treated *n* = 48, Injected *n* = 18 except *yw* Mock T3 *n* = 17 and *yw* Virus T3 *n* = 15, mean ± SD, (*P ≤ 0.025, ***P < 0.0003). **(C)** Dcr-2 levels along time upon feeding; Mock T0 = Mock-control at time point 0; Mock T3 = Mock-control at time point 3; Virus T0 = Virus infection at time point 0; Virus T3 = Virus infection at time point 3. A.U. indicates arbitrary units. Analysis performed using the Mann-Whitney test; all groups are compared with non-treated. Non-treated *n* = 48, fed *n* = 9, mean ± SD (*P ≤ 0.025, ***P = 0.0003, ****P < 0.0001).

The data shown in Figure 5 correspond to Dcr-2 protein levels relative to the mock-infected time point 0. Therefore, we cannot differentiate if the change observed is due to an absolute Dcr-2 increase after the infection or to an absolute Dcr-2 decrease in the control flies due to the stress caused by the experimental procedure (injury in the case of injection, and starvation in the case of feeding—see modes of infection in Materials and Methods). To answer this question, we compared the absolute protein levels for each time point with the basal level of the non-injected control flies from Figure 2. Since we observed the same effect on DCV and FHV injected flies, we pooled the data of all infected flies. We then analyzed the absolute levels of Dcr-2 among strains for each time point. As expected, we did not observe any strain-dependent difference in the levels of Dcr-2 (Figure 7A), in agreement with the results reported in Figure 2. This allowed us to pool each treatment condition (mock infected, virus infected and non-injected) to analyze the absolute levels of Dcr-2 across time and independently of the fly strain. Figure 7B shows that, at time 0, Dcr-2 levels in mock-infected flies rapidly decrease compared to the non-injected control. In contrast, injection of virus increased the level of Dcr-2 protein. At 3 days post-infection, the tendency reversed with increased levels of Dcr-2 in mock-infected flies and decreased levels in virus-infected flies. The same analysis was performed for flies infected by feeding. Figure 7C shows the same trend with a prompt increase of Dcr-2 protein levels upon virus ingestion and a later decrease. We did not observe a significant decrease of Dcr-2 levels during mock infection at time point 0, but we did find a significant increase for the time point 3. This indicates that starvation, as well as injection, induces a change in the regulation of Dcr-2.

Altogether, these results put in evidence a change of Dcr-2 protein levels that is not only dependent on virus infection but also on infection procedure-related stress. While virus infection rapidly induces an increment of Dcr-2 protein concentration, the infection procedure-related stress immediately decreases Dcr-2 protein levels. The increase of Dcr-2 protein during viral infection is strong enough to mask the decrease produced by stress.

## Discussion

Biological systems are composed of two features: pathways and mechanisms (22). Pathways describe the flow of entities or information along space and time; mechanisms describe indirectly the reason these pathways exist. Studying pathways or mechanisms implies studying two different things. In the case of RNAi, if we consider the hierarchical scale that includes the entire RNAi phenomenon, studying the pathway means to study the components of such pathway, the causal way they interact and the flow of these interactions until reaching the “interference” event, which it is relevant to a variety of outcomes. Studying the mechanism means to study how such a pathway works, how it is controlled and regulated, how it is induced or repressed, and for what specific outcome (22). Dcr-2 and Ago-2 are the best characterized enzymes involved in the insect antiviral siRNA pathway. Several lines of evidence demonstrated the pivotal role of this molecular process as the major antiviral response at cellular and systemic level, against both natural and unnatural *D. melanogaster* viruses (23–28). Nevertheless, we still have a scarce comprehension of the biological implication of the siRNA pathway in gene regulation (11) and in trans-kingdom communication (29–31). A comprehensive understanding of the regulation of the siRNA-mediated response upon different biotic and abiotic stimuli, from virus infection and cellular stress to environmental cues, could shed light on the global role that this pathway plays in the organism. As said by Cornish-Bowden et al. (32), “Only through the understanding of the whole can we understand the functions of the parts”.

In this work, we focused on the response of the core components of the siRNA pathway at the transcriptional and translational levels upon viral infection in *D. melanogaster* using three fly strains, two modes of infection and natural and unnatural viral pathogens.

We observed a strain-dependent susceptibility to viral infections; *yw* flies were more susceptible to viral infections than *w*^1118^ and *Oregon-R* flies in all experimental conditions. In agreement with our previous work (21), the mortality after oral infection with DCV was lower with respect to mortality following viral injection. Previous studies reported differences in the immune response of the most common laboratory strains of *D. melanogaster* used in immunity research (33, 34). For example, Okado et al. (33) show that these strains differ in susceptibility to infection, as well as differences in bacterial load and antimicrobial peptides expression profile upon infection with *Lysteria*. In addition, upon bacterial infection with *E. coli, M. luteus*, and *E. faecalis, yw* displays higher mortality in comparison with other strains (34). Since *yw* fat storage levels decreased during bacterial infection without increases in bacterial load, the authors concluded that the *yw* strain was less tolerant to bacterial infection rather than less resistant (34). As we observed a significant increment of viral load in *yw* flies that correlated with a lower percentage of survival, we cannot advance the hypothesis of a change in tolerance. Nevertheless, several studies highlight a link between metabolism changes and viral resistance in *Drosophila* (18, 35, 36); therefore we do not exclude the involvement of metabolism as an explanation for the different survival trends we noticed among strains.

Previous studies demonstrated that viral infections trigger *Dcr-2* and *Ago-2* mRNA expression in *Apis mellifera* and *Bombus terrestris* (37), and several authors suggested that this induction might be an essential feature to deal against infections in insects (13–15). For these reasons, we hypothesized that variations in *Dcr-2* and *Ago-2* expression levels upon viral infection could explain the difference in mortality between strains. Interestingly, we did not observe changes in transcript expression levels for *Dcr-2* and *Ago-2* in any strain and for any virus and mode of infection. Our results agree with previous *D. melanogaster* studies [reviewed in (38)] that showed that RNAi genes do not alter their expression level after infection with DCV. Interestingly, a recent study in which *D. melanogaster* is used as a model to investigate Zika virus-insect interactions, showed an increased level of *Dcr-2* and *Ago-2* transcripts after infection (18). As previously showed for *A. mellifera* (19), the regulation of *Dcr-2* and *Ago-2* expression may be virus-dependent. A broader study including other model viruses from several Baltimore classes should shed light on whether the absence of changes in transcript expression levels, and mainly of induction of Dcr-2 and Ago-2 proteins, is a general response against infections with natural viruses in *D. melanogaster*.

It is well established that there is not always a direct correlation between mRNA and protein levels (39, 40), and that translational and post-translational regulation and degradation of proteins play a pivotal role in determining protein levels (41). In the current work we showed that, although their transcripts remain unchanged, Dcr-2 and Ago-2 protein levels change after injection-related stress and viral infection. Injection with a sterile solution causes a rapid Dcr-2 level decrease, detectable within minutes and possibly related to a stall in protein translation and/or an increase in protein degradation. Although the actual mechanism is still unknown, similar processes of rapid decrease in protein levels are reported in the literature (42, 43) and can be associated with gene regulation (44). For example, the stimulation with 10 ng/ml of TNF-α induces a fast degradation of IκBα (the NF-κB inhibitor) with a half-life of 16 min (44). On the contrary, we observe that injection with virus leads to an increase of Dcr-2 levels. This suggests that the flies can “sense” the presence of a virus early before the beginning of viral replication, possibly via specific pattern recognition receptors, an assumption previously hypothesized (45, 46) and recently reinforced by results showing that injection of heat-inactivated Zika virus induces certain antiviral immune mechanisms (18). The increase of protein concentration without change in gene expression may be achieved by blocking protein degradation and/or increasing protein translation in a mechanism known as “*translation on demand*” (40). This molecular process seems to play an important role in protein regulation in yeast and other organisms, including mammals (40, 47, 48). For Dcr-2, the aftermath of this increase was a progressive reduction in protein levels over time that correlates with an increase in viral replication. Two non-exclusive explanations may account for this: (1) the DCV and FHV viral suppressors of RNAi (8) trigger the degradation of Dcr-2 and Ago-2 in a similar fashion to that observed for Cricket Paralysis Virus (CrPV) (49); (2) the later activation of other unknown immunological processes to fight viral infection inhibits Dcr-2 and Ago-2 from being produced.

Several lines of evidence demonstrate that immunological responses can be virus-specific. The fact that other pathways can be more effective than RNAi in counteracting viral infection has been proposed for the case of *B. terrestris* infected with Israeli Acute Paralysis virus and Slow Bee Paralysis virus (16). We have previously demonstrated that RNAi is not necessary for the clearance of viruses after oral infection (21). Other works showed that the antiviral RNAi is ineffective against virus in the midgut of *Aedes aegypti* (50) or in certain basal metazoans (51). Altogether these results highlight the fact that other biological processes may be more effective than, or work together with, the siRNA pathway to reach an effective antiviral response.

## Conclusions

In our work we showed that the key components of the antiviral siRNA pathway in *D. melanogaster*, Dcr-2 and Ago-2, are not induced at the mRNA level upon viral infection, but that their regulation occurs at the protein level through an unknown mechanism reminiscent of translation on demand. This response is independent of the mode of infection, the virus, and the fly strain, and is not related to differences in susceptibility to viral infection among strains. We also reported the bivalent aspect of this regulation, which acts as a general gene regulation mechanism (during infection procedure-related stress) and as an antiviral response (during viral infection), possibly activated via early recognition of viral motifs by unknown pattern recognition receptors.

## Data Availability Statement

The raw data supporting the conclusions of this article will be made available by the authors, without undue reservation, to any qualified researcher.

## Author Contributions

Conceptualization: AT, VM, JM, and M-CS. Methodology and investigation: AT, VM, and JM. Resources: M-CS. Formal analysis and writing—original draft preparation: AT. Writing—review and editing: VM, JM, and M-CS.

### Conflict of Interest

The authors declare that the research was conducted in the absence of any commercial or financial relationships that could be construed as a potential conflict of interest.
