# Supplementary material for: Viral Infection and Stress Affect Protein Levels of Dicer 2 and Argonaute 2 in Drosophila melanogaster
Source: Front Immunol. 2020 Mar 4;11:362. doi: 10.3389/fimmu.2020.00362 (PMC7065269; doi:10.3389/fimmu.2020.00362)
Supplement: Supplementary file 1 [file Image_1.pdf]

A

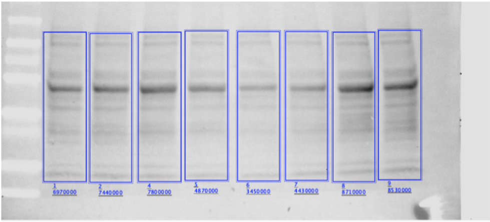

Images | Shapes

Image Name	Channel	Name	Signal	Total	Area	Bkgnd.	Type
BLOT GFMI 20...	W	1	6970000	98800000	43623	2110	Signal
BLOT GFMI 20...	W	2	7440000	97600000	43623	2070	Signal
BLOT GFMI 20...	W	4	7800000	96800000	43623	2040	Signal
BLOT GFMI 20...	W	5	4870000	93100000	43623	2020	Signal
BLOT GFMI 20...	W	6	3450000	92300000	43623	2040	Signal
BLOT GFMI 20...	W	7	4430000	94700000	43623	2070	Signal
BLOT GFMI 20...	W	8	8710000	102000000	43623	2130	Signal
BLOT GFMI 20...	W	9	8530000	104000000	43623	2200	Signal

B

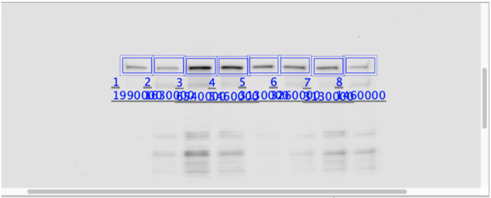

Images | Shapes

Image Name	Channel	Name	Signal	Total	Area	Bkgnd.	Type
Chemi 4m40s ...	W	1	1990000	2240000	684	356	Signal
Chemi 4m40s ...	W	2	1630000	1860000	684	334	Signal
Chemi 4m40s ...	W	3	6540000	7300000	684	1120	Signal
Chemi 4m40s ...	W	4	5460000	6040000	684	844	Signal
Chemi 4m40s ...	W	5	3130000	3270000	684	211	Signal
Chemi 4m40s ...	W	6	3260000	3700000	684	641	Signal
Chemi 4m40s ...	W	7	3130000	3450000	684	472	Signal
Chemi 4m40s ...	W	8	1460000	1680000	684	326	Signal

Supplementary Figure 1: Total protein and Dcr-2 quantifications in *yw* flies orally infected with DCV. (**A**) Total protein in a SDS-PAGE gel was transferred to a nitrocellulose membrane and visualized using Molecular Imager Gel Doc XR+ (BIO-RAD). From the left: mock 0, mock 1, mock 3, mock 6, DCV 0, DCV 1, DCV 3, DCV 6. (**B**) Western Blot anti-Dcr-2. Expected molecular weight 197 kDa. Band intensity inside the blue rectangle was estimated using ImageStudioLite (LI-COR Biosciences).
