# Supplementary material for: Viral Infection and Stress Affect Protein Levels of Dicer 2 and Argonaute 2 in Drosophila melanogaster
Source: Front Immunol. 2020 Mar 4;11:362. doi: 10.3389/fimmu.2020.00362 (PMC7065269; doi:10.3389/fimmu.2020.00362)
Supplement: Supplementary file 2 [file Image_2.pdf]

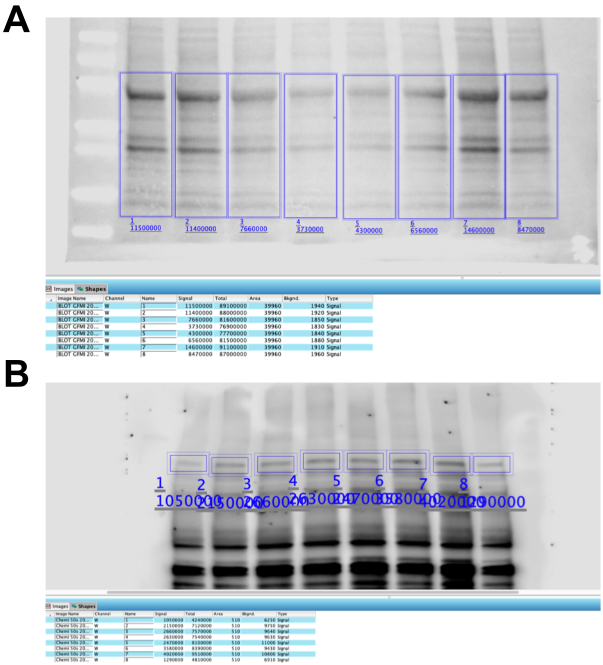

	Image Name	Channel	Name	Signal	Total	Area	Bkgnd.	Type
	BLOT GFMI 20...	W	1	11500000	89100000	39960	1940	Signal
	BLOT GFMI 20...	W	2	11400000	88000000	39960	1920	Signal
	BLOT GFMI 20...	W	3	7660000	81600000	39960	1850	Signal
	BLOT GFMI 20...	W	4	3730000	76900000	39960	1830	Signal
	BLOT GFMI 20...	W	5	4300000	77700000	39960	1840	Signal
	BLOT GFMI 20...	W	6	6560000	81500000	39960	1880	Signal
	BLOT GFMI 20...	W	7	14600000	91100000	39960	1910	Signal
	BLOT GFMI 20...	W	8	8470000	87000000	39960	1960	Signal

	Image Name	Channel	Name	Signal	Total	Area	Bkgnd.	Type
	Chemi 50s 20...	W	1	1050000	4240000	510	6250	Signal
	Chemi 50s 20...	W	2	2150000	7120000	510	9750	Signal
	Chemi 50s 20...	W	3	2660000	7570000	510	9640	Signal
	Chemi 50s 20...	W	4	2630000	7540000	510	9630	Signal
	Chemi 50s 20...	W	5	2470000	8100000	510	11000	Signal
	Chemi 50s 20...	W	6	3580000	8390000	510	9430	Signal
	Chemi 50s 20...	W	7	4020000	9510000	510	10800	Signal
	Chemi 50s 20...	W	8	1290000	4810000	510	6910	Signal

Supplementary Figure 2: Total protein and Ago-2 quantifications in *white*^{1118} flies injected with FHV. (**A**) Total protein in a SDS-PAGE gel was transferred to a nitrocellulose membrane and visualized using Molecular Imager Gel Doc XR+ (BIO-RAD). From the left: mock 0, mock 1, mock 2, mock 3, DCV 0, DCV 1, DCV 2, DCV 3. (**B**) Western Blot anti-Ago-2. Expected molecular weight 137 kDa. Band intensity inside the blue rectangle was estimated using ImageStudioLite (LI-COR Biosciences).
