# Supplementary material for: Viral Infection and Stress Affect Protein Levels of Dicer 2 and Argonaute 2 in Drosophila melanogaster
Source: Front Immunol. 2020 Mar 4;11:362. doi: 10.3389/fimmu.2020.00362 (PMC7065269; doi:10.3389/fimmu.2020.00362)
Supplement: Supplementary file 3 [file Table_1.pdf]

DCV Injections

Exp	Strain	Treatment	tpi	TDID50/fly	Exp	Strain	Treatment	tpi	TDID50/fly	Exp	Strain	Treatment	tpi	TDID50/fly
1	*Oregon-R*	Mock	T0	0,00E+00	2	*Oregon-R*	Mock	T0	0,00E+00	3	*Oregon-R*	Mock	T0	0,00E+00
			T1	0,00E+00				T1	0,00E+00				T1	0,00E+00
			T2	0,00E+00				T2	0,00E+00				T2	0,00E+00
			T3	0,00E+00				T3	0,00E+00				T3	0,00E+00
		DCV inj	T0	6,78E+01			DCV inj	T0	0,00E+00			DCV inj	T0	7,03E+00
			T1	6,78E+04				T1	2,22E+06				T1	3,95E+01
			T2	2,14E+07				T2	3,95E+07				T2	1,25E+07
			T3	2,14E+06				T3	2,07E+07				T3	3,95E+07
	w^{1118}	Mock	T0	0,00E+00		w^{1118}	Mock	T0	0,00E+00		w^{1118}	Mock	T0	0,00E+00
			T1	0,00E+00				T1	0,00E+00				T1	0,00E+00
			T2	0,00E+00				T2	0,00E+00				T2	0,00E+00
			T3	0,00E+00				T3	0,00E+00				T3	0,00E+00
		DCV inj	T0	6,78E+01			DCV inj	T0	0,00E+00			DCV inj	T0	1,25E+01
			T1	3,81E+05				T1	3,95E+06				T1	1,25E+05
			T2	1,21E+07				T2	5,27E+07				T2	5,27E+07
			T3	6,78E+07				T3	2,50E+08				T3	2,50E+08
	yw	Mock	T0	0,00E+00		*yw*	Mock	T0	0,00E+00		*yw*	Mock	T0	0,00E+00
			T1	0,00E+00				T1	0,00E+00				T1	0,00E+00
			T2	0,00E+00				T2	0,00E+00				T2	0,00E+00
			T3	0,00E+00				T3	0,00E+00				T3	0,00E+00
		DCV inj	T0	6,78E+01			DCV inj	T0	0,00E+00			DCV inj	T0	0,00E+00
			T1	3,81E+07				T1	3,95E+07				T1	2,22E+07
			T2	5,00E+08				T2	5,27E+08				T2	9,37E+08
			T3					T3					T3	

Supplementary Table 1: Viral titers for *Oregon-R* , w^{1118} , and *yw* injected with DCV. Three biological replicates are shown.

DCV Oral infections

Exp	Strain	Treatment	tpi	TDID50/fly	Exp	Strain	Treatment	tpi	TDID50/fly	Exp	Strain	Treatment	tpi	TDID50/fly
1	*Oregon-R*	Mock	T0	0,00E+00	2	*Oregon-R*	Mock	T0	0,00E+00	3	*Oregon-R*	Mock	T0	0,00E+00
			T1	0,00E+00				T1	0,00E+00				T1	0,00E+00
			T3	0,00E+00				T3	0,00E+00				T3	0,00E+00
			T6	0,00E+00				T6	0,00E+00				T6	0,00E+00
		DCV Feeding	T0	6,59E+05			DCV Feeding	T0	4,45E+05			DCV Feeding	T0	3,70E+05
			T1	6,59E+04				T1	2,50E+04				T1	3,70E+04
			T3	6,59E+06				T3	2,50E+07				T3	3,70E+08
			T6	1,17E+02				T6	1,41E+07				T6	1,17E+01
	w^{1118}	Mock	T0	0,00E+00		w^{1118}	Mock	T0	0,00E+00		w^{1118}	Mock	T0	0,00E+00
			T1	0,00E+00				T1	0,00E+00				T1	0,00E+00
			T3	0,00E+00				T3	0,00E+00				T3	0,00E+00
			T6	0,00E+00				T6	0,00E+00				T6	0,00E+00
		DCV Feeding	T0	1,17E+06			DCV Feeding	T0	7,91E+04			DCV Feeding	T0	6,59E+04
			T1	6,59E+05				T1	7,91E+06				T1	3,70E+06
			T3	2,08E+07				T3	1,41E+03				T3	6,59E+07
			T6	6,59E+07				T6	2,50E+02				T6	3,70E+07
	yw	Mock	T0	0,00E+00		*yw*	Mock	T0	0,00E+00		*yw*	Mock	T0	0,00E+00
			T1	0,00E+00				T1	0,00E+00				T1	0,00E+00
			T3	0,00E+00				T3	0,00E+00				T3	0,00E+00
			T6	0,00E+00				T6	0,00E+00				T6	0,00E+00
		DCV Feeding	T0	6,59E+04			DCV Feeding	T0	2,50E+05			DCV Feeding	T0	3,70E+05
			T1	2,08E+07				T1	4,45E+04				T1	6,59E+03
			T3	1,17E+08				T3	4,45E+06				T3	1,17E+08
			T6	3,70E+07				T6	7,91E+06				T6	1,17E+05

Supplementary Table 1: Viral titers for *Oregon-R* , w^{1118} , and *yw* orally infected with DCV. Three biological replicates are shown.

FHV Injections

Exp	Strain	Treatment	tpi	TDID50/fly	Exp	Strain	Treatment	tpi	TDID50/fly	Exp	Strain	Treatment	tpi	TDID50/fly
1	*Oregon-R*	Mock	T0	0,00E+00	2	*Oregon-R*	Mock	T0	0,00E+00	3	*Oregon-R*	Mock	T0	0,00E+00
			T1	0,00E+00				T1	0,00E+00				T1	0,00E+00
			T2	0,00E+00				T2	0,00E+00				T2	0,00E+00
			T3	0,00E+00				T3	0,00E+00				T3	0,00E+00
		DCV inj	T0	1,41E+03			DCV inj	T0	1,97E+02			DCV inj	T0	2,50E+02
			T1	2,50E+04				T1	1,97E+03				T1	2,50E+06
			T2	7,91E+05				T2	6,22E+05				T2	7,91E+06
			T3	4,45E+07				T3	6,22E+06				T3	4,45E+08
	w^{1118}	Mock	T0	0,00E+00		w^{1118}	Mock	T0	0,00E+00		w^{1118}	Mock	T0	0,00E+00
			T1	0,00E+00				T1	0,00E+00				T1	0,00E+00
			T2	0,00E+00				T2	0,00E+00				T2	0,00E+00
			T3	0,00E+00				T3	0,00E+00				T3	0,00E+00
		DCV inj	T0	1,41E+02			DCV inj	T0	3,50E+02			DCV inj	T0	2,50E+02
			T1	4,45E+04				T1	6,22E+03				T1	2,50E+05
			T2	4,45E+05				T2	6,22E+05				T2	4,45E+06
			T3	2,50E+07				T3	6,22E+06				T3	7,91E+08
	yw	Mock	T0	0,00E+00		*yw*	Mock	T0	0,00E+00		*yw*	Mock	T0	0,00E+00
			T1	0,00E+00				T1	0,00E+00				T1	0,00E+00
			T2	0,00E+00				T2	0,00E+00				T2	0,00E+00
			T3	0,00E+00				T3	0,00E+00				T3	0,00E+00
		DCV inj	T0	1,41E+02			DCV inj	T0	1,11E+02			DCV inj	T0	1,41E+03
			T1	2,50E+05				T1	3,50E+04				T1	2,50E+06
			T2	4,45E+07				T2	3,50E+05				T2	4,45E+09
			T3	2,50E+09				T3	1,11E+08				T3	4,45E+09

Supplementary Table 1: Viral titers for *Oregon-R* , w^{1118} , and *yw* injected with FHV. Three biological replicates are shown.
